# Emerging data supporting stromal cell therapeutic potential in cancer: reprogramming stromal cells of the tumor microenvironment for anti-cancer effects

**DOI:** 10.20892/j.issn.2095-3941.2020.0133

**Published:** 2020-12-15

**Authors:** Armel Hervé Nwabo Kamdje, Paul Faustin Seke Etet, Richard Tagne Simo, Lorella Vecchio, Kiven Erique Lukong, Mauro Krampera

**Affiliations:** 1 Department of Biomedical Sciences, University of Ngaoundere, Faculty of Science, Ngaoundere 454, Cameroon; 2 Department of Physiological Sciences and Biochemistry, University of Ngaoundéré, Garoua 454, Cameroon; 3 Center for Sustainable Health and Development, Garoua 454, Cameroon; 4 Department of Biochemistry, Microbiology & Immunology, University of Saskatchewan, College of Medicine, Saskatoon SK S7N 5E5, Canada; 5 Department of Medicine, University of Verona, Section of Hematology, Stem Cell Research Laboratory, Verona 37134, Italy

**Keywords:** Stromal cells, tumorigenic effects, anti-cancer effects, tumor microenvironment, reprogramming

## Abstract

After more than a decade of controversy on the role of stromal cells in the tumor microenvironment, the emerging data shed light on pro-tumorigenic and potential anti-cancer factors, as well as on the roots of the discrepancies. We discuss the pro-tumorigenic effects of stromal cells, considering the effects of tumor drivers like hypoxia and tumor stiffness on these cells, as well as stromal cell-mediated adiposity and immunosuppression in the tumor microenvironment, and cancer initiating cells’ cellular senescence and adaptive metabolism. We summarize the emerging data supporting stromal cell therapeutic potential in cancer, discuss the possibility to reprogram stromal cells of the tumor microenvironment for anti-cancer effects, and explore some causes of discrepancies on the roles of stromal cells in cancer in the available literature.

## Introduction

Mesenchymal stromal cells (MSCs) are a heterogeneous mesenchymal cell population, commonly collected from the bone marrow (BM), fat, and other tissues, that includes multipotent stem cells capable of differentiating into a number of mesenchymal tissues, and thus can contribute to tissue repair. MSCs are positive for membranes CD105, CD73, and CD90, and negative for CD14, CD19, CD31, CD34, CD45, and HLA-DR^1,2^. MSC differentiation potential includes various cell types of the mesodermal lineage, such as fibroblasts, adipocytes, endothelial cells, myocytes, chondrocytes, and osteoblasts^1^, while non-mesodermal differentiation, such as into neural, hepatic, pancreatic, and gastric cells, is still debated^3–6^. However, MSCs reside not only in the stroma of various tissues and organs, but also in the tumor microenvironment, where their role has been clarified recently^7,8^. Overall, a huge body of evidence supports that MSCs can promote tumorigenic processes, such as: (i) angiogenesis, neovascularization and formation of cancer stem cell (CSC) niche; (ii) malignant transformation, maintenance of cancer cells, and metastasis formation; as well as (iii) cancer cell stemness and chemoresistance to anti-cancer drugs^9,10^.

On the other hand, MSCs have become a key tool in tissue engineering and regenerative medicine, because they are easily collected and have the ability to migrate and home into damaged tissues. Here, they: (i) interact with the microenvironment to drive tissue repair; (ii) differentiate into the specific affected cell types to restore or replace damaged tissues; and (iii) rescue organ functions, thanks to their high proliferation, adhesion, migration, differentiation, and immunoregulatory properties^11–13^. Notably, MSC secretome includes numerous factors favoring tissue repair, such as angiopoietin-1, vascular endothelial growth factor (VEGF), transforming growth factor-beta (TGF-β), fibroblast growth factor (FGF), hepatocyte growth factor (HGF), epidermal growth factor (EGF), platelet-derived growth factor (PDGF), granulocyte-colony stimulating factor (G-CSF)^14–19^, as well as other soluble factors, such as interleukin-6 (IL-6), IL-12, C-X-C motif chemokine 8 (CXCL8), CXCL9, CXCL16, C-C chemokine ligand 20 (CCL20), CCL25, and monocyte chemoattractant protein-3 (MCP-3)^20–23^.

Herein, we provide an overview of recent data suggesting that the pro-tumorigenic effects of MSCs as well as MSC-derived cancer-associated fibroblasts (CAFs) are the consequence of a process of cell reprogramming driven by the tumor microenvironment. We also discuss the emerging reports suggesting approaches to reprogram these cells to mediate anti-tumor effects *in vivo*, as well as data supporting the existence of stromal cells restraining cancer growth in the tumor microenvironment.

## Pro-tumorigenic effects of stromal cells

### Immune abnormalities

#### Immunosuppression

It is well established that MSCs are major drivers of the typical immunomodulation observed in a solid tumor microenvironment. For instance, a recent study using MSCs expanded from BM and prostate cancer tissue from independent donors showed that tumor-infiltrating MSCs are major drivers of the immunosuppressive tumor microenvironment in prostate cancer^24^. The authors reported the ability of prostate cancer-infiltrating MSCs to suppress T-cell proliferation through immunosuppressive properties comparable to canonical BM-derived MSCs. The suppression of proliferation mediated by prostate cancer-infiltrating MSCs was dose-dependent, and the expressions of programmed cell death ligand 1 (PD-L1) and programmed cell death ligand 2 (PD-L2) were upregulated on T cells in the presence of interferon-γ (IFN-γ) and tumor necrosis factor-α (TNF-α)^24^. In another study, the transcriptome analysis of MSCs from multiple myeloma (MM) patients revealed constitutive abnormalities in immune system activation, cell cycle progression, and osteoblastogenesis that were maintained even in the absence of tumors cells, thus strongly suggesting that MSCs may contribute to the immune evasion and bone lesions frequently found in MM^25^. MSCs shape the myelodysplastic syndrome microenvironment at least in part by inducing suppressive monocytes dampening natural killer (NK) cell function^26^. Moreover, MSCs participate in oral mucosa carcinogenesis by increasing immunosuppressive functions on T-cell proliferation; tumorigenesis of tumor-resident MSCs correlated with higher expression of cellular proliferative status indicator Ki67^27^. Interestingly and on the same hand, the CXCL8 supporting the survival and proliferation of acute myeloid leukemia (AML) cells *via* the phosphatidylinositol 3-kinase (PI3K)/protein kinase B (AKT) signaling pathway in the affected BM microenvironment would be mainly secreted by MSCs^28^.

#### Cellular senescence

The normal aging process and various age-related diseases, including some cancers, are marked by a chronic low-grade inflammation (“inflammaging”) and cellular senescence (“immunosenescence”). The role of MSC immunomodulation in shaping a senescent microenvironment in a broad spectrum of human malignancies, especially tumorigenesis, has been documented extensively^29,30^. For instance, gastric cancer cell-derived exosomes (extracellular vesicles) affect the immunomodulatory functions of MSCs by activating the nuclear factor-kappa B (NF-κB) signaling pathway, which in turn mediates support to tumor growth by maintaining the inflammatory environment and enhancing the ability of MSCs to activate immune cells^31^. AML blasts induce a senescence-associated secretory phenotype (SASP) in BM stromal cells through a p16INK4a-dependent mechanism, which encompasses the irreversible arrest of cell proliferation and the secretion of a set of chemokines, proinflammatory cytokines, and growth factors^32^. Similarly, some authors reported the alteration of cellular and immune-related properties of BM-derived MSCs (BM-MSCs) and macrophages through the release of exosomes from K562 chronic myeloid leukemia cell line; exosome concentration in BM-MSCs correlated with the enhanced expression of Dickkopf-related protein 1 (DKK1), wnt5a, CXCL12, IL-6, TGF-β, and TNF-α^33^. Furthermore, senescent breast luminal cells promoted carcinogenesis by activating CAFs through the inflammatory cytokine IL-8^34^. BM stromal cells from patients with myelodysplastic syndrome display a senescence phenotype induced by S100A9-induced Toll-like receptor 4 (TLR4), NLR family pyrin domain containing 3 (NLRP3) inflammasome activation, and IL-1β secretion^35^. TLR4 signaling was also reported to drive MSC commitment to promote tumor microenvironment transformation in MM^36^.

### Cancer-associated metabolic changes

Various authors have reported the involvement of microenvironmental stromal cells in cancer-associated metabolic changes supporting tumorigenic processes. Adaptive metabolic plasticity, i.e., tumor-initiating cell ability to switch between oxidative phosphorylation and glycolysis, depending on reactive oxygen species, hypoxia, and glucose availability in the tumor microenvironment, confers a survival advantage to malignant cells in breast cancer, thus representing a potential target for anti-cancer therapy^37^. Notably, the overexpression of O-GlcNAc transferase (OGT), an enzyme involved in tumor-initiating cell-mediated rewiring of energy metabolism, increases CSC populations and mammosphere formation *in vitro* and *in vivo*. The pharmacological or genetic inhibition of OGT induces a potent reduction of mammosphere formation, as well as CD44H/CD24L, ALDH+, and NANOG+ tumor-initiating cell populations in breast cancer cells^38^. These observations confirm that the inhibition of adaptive metabolic plasticity may serve as a therapeutic strategy to regulate tumor-initiating activity in breast cancer.

Similarly, pancreatic cancer cells utilize “metabolic reprogramming”, through the enhancement of glycolysis with increased lactate production and glycolytic enzyme overexpression, to satisfy their energy demand and support malignant behaviors, despite a hypoxic and nutrient-deficient microenvironment^39^. A study in bevacizumab-resistant glioblastoma suggested that chemoresistance in cancer cells inside the hypoxic microenvironment occurs through: (i) metabolic reprogramming, with suppressed oxidative phosphorylation and upregulated glycolysis; (ii) perivascular invasiveness along remaining blood vessels in a VEGF- and neo-angiogenesis-independent manner; and (iii) enrichment of tumor-initiating stem cells residing in the perivascular niche close to residual blood vessels^40^. In addition, exosomes from glioma cells induced a tumor-like phenotype in MSCs by activating glycolysis^41^.

In 2019, a report from Lung and colleagues^42^ showed that the expression of estrogen receptor (ER)-α, the target of endocrine therapies in breast cancer that is expressed by most metastatic breast cancer cells, is regulated by the BM microenvironment. In this study, the induction of estrogen receptor 1 (ESR1) mRNA and ER protein downregulation, through a mitogen- activated protein kinase (MAPK)-independent mechanism, was achieved by the treatment of breast cancer cells with conditioned culture media from either cancer-activated BM stromal cells or HS5 BM stromal cell line. In addition, thyroid hormones, which are well-established pro-tumorigenic players, may stimulate tumor growth and neovascularization in various solid cancers by activating MSCs through a non-classical integrin αvβ3 signaling^43^. Moreover, the EGF-like superfamily member EGFL6, playing an important role during embryonic development without any effect on wound healing, mediates a crosstalk between cancer and stromal cells to induce stemness and epithelial–mesenchymal transition (EMT) (EMT is an important tumorigenic mechanism where epithelial cells become MSCs by losing their cell polarity and adhesion ability, and gaining migratory and invasive properties) in breast cancer cells *in vitro*, thus promoting tumor growth *in vivo*^44^.

### Cancer-promoting CAFs

CAFs play a pivotal role in cancer progression, partially through signaling molecules that may represent potential therapeutic strategies for cancer treatment. For instance, the overexpression of the potential prognostic factor, heat shock factor 1 (HSF1), promotes EMT, proliferation, migration, and invasion in Cal27 cells. The presence of CAFs expressing CD10 and GPR77 correlates with poor survival and chemoresistance in lung and breast cancer patients, and these CAFs supported cancer stemness and promoted cancer formation and chemoresistance in patient-derived xenografts^45^. In addition, HSF1 stimulates tumor growth in nude mice and its expression significantly correlates with poor overall survival and prognosis in patients with oral squamous cell carcinoma^46^. Intracellular Notch1 signaling in CAFs inversely controls stromal regulation of the stemness and plasticity of CSCs in melanoma, acting as a molecular switch modulating tumor heterogeneity and aggressiveness^47^.

Strong evidence that stromal microenvironment shapes the intratumoral architecture in pancreatic tumors was shown by a study using single-cell RNA, protein analysis, and high-content digital imaging of RNA *in situ* hybridization to assess the role of stromal CAFs in the modulation of heterogeneity in pancreatic ductal adenocarcinoma (PDA) models^48^. The authors identified significant single-cell population shifts toward proliferative phenotypes and invasive EMT linked to MAPK and signal transducer and activator of transcription 3 (STAT3) signaling, which contributed to intratumoral heterogeneity in tumor glands and to differences in stromal abundance and clinical outcome. Furthermore, a study addressing the ability of mesenchymal HT1080 fibrosarcoma cell line to switch to amoeboid motility (migration plasticity) revealed that pharmacological or RNA interference (RNAi)-mediated downregulation of the Arp2/3 complex or decrease of adhesiveness to its substrate induced the transition from a lamellipodium-rich to a blebbing phenotype in fibrosarcoma cells, but not in normal subcutaneous fibroblasts^49^. Interestingly, still in this study, a significant fraction of fibrosarcoma cells expressing the blebbing phenotype exhibited stem cell-like features, such as increased efflux of Hoechst-33342 and CD133, Oct4, Sox2 and Nanog expression, and demonstrated an increased ability to switch to a bleb-rich amoeboid phenotype in three-dimensional (3D) collagen gels^49^.

## Stromal cells’ therapeutic potential in cancer

### Damage repair after chemotherapy

Various reports support the therapeutic potential of MSCs in cancer, also for repairing damaged tissues after chemotherapy^50,51^. For instance, human adipose-derived MSCs displayed repairing properties in damaged thymus following chemotherapy in mouse models of blood cancer^52^. Mice showed improvements in the thymic structure and functions, as shown by the proportion of circulating and splenic regulatory T (Treg) cells and the recovery of T-cell subpopulations.

### MSCs slowing tumor progression

In a study involving both human colorectal cancer cells and immunocompetent rat models of colorectal carcinogenesis, the treatment with BM-derived MSCs interfered with colon cancer progression. The effect was partially due to the modulation of the tumor microenvironmental immune effector cells, such as Tregs, CD8+ cells, and NK cells. In addition, there was evidence of Th17 cell activity restoration, macrophage reprogramming into regulatory cells performing phagocytosis with reduced production of proinflammatory cytokines, a 50% decrease in the infiltration rate of CD68+ cells, and a two-fold increase of CD3+ cells^53^. Two microRNAs, i.e., small non-coding RNA molecules silencing post-transcriptional regulation of gene expression, associated with the capacity of MSCs to attenuate cancer growth were identified, namely microRNA 150 (miR-150) and miR-7. Similarly, human BM-MSC-derived exosomes overexpressing miR-34a inhibited glioblastoma development^54,55^. In another study, intra-BM but not systemic administration of BM-MSCs from healthy donors reduced tumor burden and prolonged survival of the leukemia-bearing mice^54^. In this study, the MSC senescence observed during disease progression was stopped and the BM microenvironment was restored, with functional recovery of host myelopoiesis and improvement of thrombopoiesis. Moreover, in a bioluminescence imaging study monitoring the effects of human umbilical cord-derived MSCs in mouse hepatoma tumor models with H7402 cell line, the MSC microenvironment effectively inhibited the growth of cancer cells^56^.

### Cancer-restraining CAFs

Different studies, both clinical and in mouse models, suggest that there may exist at least two populations of MSC-derived CAFs, i.e., cancer-promoting CAFs, discussed already, and cancer-restraining CAFs^57^. However, the identity of cancer-restraining CAFs remains poorly investigated, due to the lack of markers. Interestingly, a cell subpopulation with tumor inhibitory functions was isolated and characterized in a cancer metastasis microenvironment by using stromal cell lines derived from the central nervous system (CNS) metastasis of breast and lung cancer patients^58^. Interestingly, these cells were quite homogenous, expressed high levels of collagen, and displayed gene expression signatures of CAFs, MSCs, and EMT^58^. Mizutani and colleagues^59^ reported the glycosylphosphatidylinositol-anchored protein Meflin as a potential marker of cancer-restraining CAFs. These authors observed that the tissue infiltration of Meflin-positive CAFs correlated with a favorable patient outcome in PDA. By contrast, Meflin deficiency or low expression resulted in a markedly faster tumor progression in a PDA mouse model, and either the overexpression of Meflin in CAFs or the delivery of a Meflin-expressing lentivirus into the tumor stroma was sufficient to suppress the growth of xenograft tumors^59^. This new marker paves the way for isolation and further characterization of CAFs exerting anti-tumoral effects.

## Stromal cells follow the program dictated by their microenvironment

### Stromal cells’ programming by tumor microenvironment

#### Effects of the tumor microenvironment on stromal cells

Early studies addressing the composition of the tumor microenvironment reported an atypical cellular and molecular microenvironment supporting carcinogenesis and chemoresistance^60,61^. Recently, Coffman and colleagues^62^ reported that ovarian carcinoma-associated MSCs, which are critical stromal progenitor cells promoting tumor cell growth, cancer stemness, and chemoresistance, arise from a process of tumor-mediated reprogramming of local tissue MSCs. This study also provided strong evidence that tumor-mediated MSC conversion is tissue- and cancer-type dependent, and requires tumor-secreted factors and hypoxia^62^. Breast tumor microenvironment transforms naive MSCs into tumor-forming cells in nude mice; in addition, MSCs pre-exposed to a conditioned medium or purified exosomes derived from breast cancer cells (MDA-MB-231) form a tumor-like mass rich in stromal tissue by 14 weeks when injected into mammary glands of nude mice^63^. Similarly, CCL5 secreted by classic Hodgkin lymphoma cells recruits MSCs and monocytes and enhances MSC proliferation and CCL5 secretion; conditioned medium from these MSCs increases tumor cell growth and monocyte migration^64^. Exosomes derived from chronic myeloid leukemia cells altered the cellular and immune-related properties of BM-MSCs and macrophages *in vitro*^33^. Moreover, the expression of gene signatures and mesenchymal shift in quiescent glioblastoma cells, a source of tumor recurrence in highly malignant glioblastoma, was observed following their interactions with niche microenvironment^65^. Reciprocal reprogramming of CSCs and associated MSCs may promote tumor progression in gastric cancer^66^. Consequently, unraveling the signaling molecules involved in pro-tumorigenic crosstalks between MSCs and tumor environment may lead to novel targets for inducing cancer regression and elimination.

Interestingly, asporin, a factor secreted by MSCs following cellular interactions within the tumor microenvironment, alters the tumor microenvironment and inhibits MSC differentiation to drive metastatic progression through CD49d/CD29 signaling^67^. MSCs promotes the progression of gastric cancer cells through the release of CXCL16, which activates STAT3-mediated expression of Ror1 in the cancer cells^68^. Dabbah and colleagues reported that microvesicles derived from BM-MSCs of MM patients increase the tumorigenicity of MM cells^69^. In this study, CD49d and CD29 integrin overexpression in MM-MSC microvesicles correlated with patient staging and response to treatment; the concomitant inhibition of these molecules resulted in reduced uptake of MM-MSC microvesicles (but not normal donor MSC microvesicles), inhibition of MM cell signaling, expression of aggressiveness markers, and enhanced response to chemotherapy^69^. This study also suggested that the reciprocal interactions of malignant cells and MSCs in breast cancer microenvironment may result in the transformation of naive MSCs into cells capable of forming explants in nude mice. Notably, pre-metastatic niche in distant organs may be created, at least in part, by the transfer to stromal cells, such as peritoneal mesothelial cells (PMCs), fibroblasts, and endothelial cells, of tumor-derived extracellular vesicles secreted by tumor-associated macrophages (TAMs) into the blood^70^. STAT4 overexpression in gastric cancer cells makes normal fibroblasts acquire CAF-like features *via* activating the wnt/β-catenin pathway^71^. In addition, Guo and colleagues^72^ (in 2019) addressed the potential roles and mechanisms of long non-coding RNAs in CSC-like properties and EMT in non-small cell lung cancer (NSCLC) using Western blot, quantitative reverse transcription polymerase chain reaction (RT-PCR), colony formation, transwell migration, and wound healing assays in A549 and H1299 human NSCLC cell lines, L9981 and 95D highly metastatic cell lines, and NL9980 and 95C low-metastatic cell lines. These authors observed that knockdown of long non-coding RNA linc-ITGB1 inhibited the expression of various markers of cancer stemness and CSC formation by reducing the expression of the EMT-related transcription factor Snail. Overexpression of Snail reversed the inhibitory effects of linc-ITGB1 knockdown^72^.

#### Role of the extracellular matrix

Emerging data strongly suggest that tumor extracellular matrix (ECM) and related factors contribute to the controversial role of stromal cells in the tumor microenvironment. For example, after showing that MM cells, cocultured with BM-MSCs, comodulated the phenotype of MM cells in an MAPKs/translation initiation (TI)-dependent manner, Ibraheem and colleagues^73^ reported that even the decellularized ECMECM of BM-MSCs from MM patients was able to induce comparable pro-tumorigenic effects. A number of changes in microRNAs was shown affecting the MM phenotype and the activation of MAPK/TI, EMT, proliferation, and CXCR4, with a role for BM-MSC secretomes and microvesicles. On the other hand, the decellularized ECM of BM-MSCs from normal donors mediated anti-cancer effects, including a rapid and persistent decrease in MAPK/TI activation, proliferation, cell count, viability, migration, and invasion^73^. These authors also provided evidence for a synergism between the ECM and microvesicles in the modulation of MM cell response to chemotherapy as well as in the hierarchy and interdependence of MAPKs/TI/autophagy/phenotype cascade. In addition, extracellular vesicles released by monocytes from chronic myelomonocytic leukemia patients are sufficient to confer a procoagulant state through a tissue factor-dependent mechanism mediated by MSCs^74^.

Matrix metalloproteinase-9 (MMP-9) produced by leukemia cells facilitates tumor progression *via* remodeling of the ECM of the BM microenvironment, and MMP-9 deficiency in the BM microenvironment reduces leukemia-initiating cells and prolongs survival of mice with BCR-ABL1-positive B-cell acute lymphoblastic leukemia (B-ALL)^75^. Similarly, senescent MSCs actively remodel the surrounding ECM to drive breast cancer cells to a more invasive phenotype^76^. Interestingly, 3D culture studies with cancer and stromal cells in ECM, incorporating multiplex quantitative analysis method, may reveal the major signaling molecules and mechanisms driving the pro- and anti-cancer interactions, providing new therapeutical targets^77,78^. Notably, in a recent study using a similar approach in hepatocellular carcinoma (HCC), cell repopulation of cirrhotic scaffolds showed a unique up-regulation of genes related to EMT and TGF-β signaling as well as a high concentration of endogenous TGF-β1 in comparison to healthy scaffolds and TGF-β1-induced phosphorylation of canonical proteins Smad2/3^79^. This study characterized the inherent features of ECM micro-environment from human cirrhotic liver as key pro-carcinogenic components in HCC development. Similarly, MSCs cocultured with colorectal cancer cells showed increased invasiveness and proliferative abilities due to increased TGF-β1 and decreased p53 levels^80^. TGF-β1 promoted the migration and invasion of HCT116 and HT29 colorectal cancer cells, and induced the differentiation of MSCs into CAFs through a Janus kinase (JAK)/STAT3 signaling-dependent mechanism^81^. Long-term coculture of human MDA-MB-231 breast cancer cells with normal human MSCs was associated with the formation of 3D tumor spheroids *in vitro*, with a 14-fold enhanced expression of the breast tumor marker urokinase plasminogen activator (uPA)^82^.

#### Hypoxia and tumor stiffness

Earlier reports suggested that hypoxia-inducible factor 1 (HIF-1) may link hypoxia, inflammation, and cancer^83,84^. In addition, recently, stromal cells were reported as mediators of the pro-tumorigenic effects of hypoxia and tumor stiffness, which are known elements of the solid tumor microenvironment promoting tumor survival, progression, and metastasis. Osteopontin, a hypoxia-driven phosphorylated glycoprotein, may promote stem cell-like properties and EMT in pancreatic cancer cells by activating the integrin αvβ3-Akt/Erk-forkhead box protein M1 (FOXM1) signaling in a paracrine manner^85^. Microvesicles derived from human BM-MSCs support human osteosarcoma (U2OS) cell growth under hypoxia *in vitro* and *in vivo* through PI3K/AKT and HIF-1α-dependent mechanisms^86^. Similarly, hypoxic BM stromal cells-derived exosomal miRNAs promote metastasis of lung cancer cells *via* STAT3-induced EMT in an *in vivo* mouse syngeneic tumor model^87^. Moreover, exosomal miRNAs from hypoxic BM-MSCs promoted lung cancer cell metastasis *via* STAT3-induced EMT^88^ and hypoxia-conditioned MSCs promote HCC progression through yes-associated protein (YAP)-mediated lipogenesis reprogramming^56^, further suggesting that targeting the communication between MSC and cancer cells may be a potential target for anti-tumor therapy.

Interactions of cancer cells and stromal cells in a hypoxic microenvironment drive EMT through NOTCH and c-MET signaling, and induce immunosuppressive response within the microenvironment in PDA, a fatal disease with limited response to both immunotherapy and cytotoxic chemoradiotherapy^89^. A study addressing the end-stage myeloma cell mobilization from the BM into peripheral blood revealed that hypoxic BM niches, together with a pro-inflammatory microenvironment resulting from the interactions between tumor cells and BM stromal cells, induce an arrest in proliferation that forces tumor cells to circulate into the peripheral blood to seek other BM niches^90^. Hypoxia-induced EMT has been shown with a 17-gene panel aimed at assessing NSCLC prognosis^91^. Similarly, hypoxia-induced acquisition of CSC features in lung cancer cells occurs through CXCR4 activation^92^. In addition, the retention factor in the endoplasmic reticulum (RER1) enhances carcinogenesis and stemness of pancreatic cancer^93^. Finally, glioblastoma stem-like cell (GSC) phenotype, the worst prognostic marker of glioblastoma, persists partially due to the hypoxic microenvironment-dependent release of extracellular adenosine, thus promoting cell migration, invasion, and tumor recurrence through the activation of the A3 adenosine receptor (A3AR)^94,95^.

MSC-derived CAFs were reported as the link between biophysical forces and pro-metastatic signaling in colon cancer, as they respond to increased stiffness of the tumor microenvironment by the activation of the signaling mediated by TGF-β family members and activin A, a strong pro-metastatic cytokine^95^. In addition, Saforo and colleagues^96^ described an *in vitro* cell culturing system incorporating elements of the *in vivo* lung environment, including physiological hypoxia (5% O_2_) and lung fibroblast-derived ECM. Through this culture system, a rapid expansion of stromal progenitors from patient’s lung tumor resections was achieved; these progenitor cells retained the secretion of factors associated with cancer progression, the expression of pluripotency markers, and the ability to enhance tumor cell growth and metastasis^96^.

#### Adiposity

It is well established that fat tissue overgrowth in obesity promotes tumor progression^97–99^. Su and colleagues^100^ compared lean and obese mice grafted with prostate tumors and showed that obesity promotes EMT in cancer cells and tumor invasion into the surrounding fat tissue. In this study, adipose stromal cells induced EMT in prostate cancer cells and made them more migratory and chemo-resistant; by contrast, adipose stromal cell targeting suppressed both EMT and chemoresistance to docetaxel, cabazitaxel, and cisplatin chemotherapy in human prostate cancer cells^100^. Human adipose-derived MSCs promoted EMT in MCF7 breast cancer cells by cross-interacting with the TGF-β/Smad and PI3K/AKT signaling pathways, in a coculture system established to investigate the paracrine effects of MSCs on the migration and invasion potential of this aggressive breast cancer cell line^101^. In addition, a study in a xenograft model of early MM showed that bone niche switching toward a “fatty” marrow supports the development of malignant cells during carcinogenesis. In this study, MSCs mainly gave rise to adipocytes supporting tumor growth by increasing the survival and chemoresistance of malignant cells^102^.

In addition, interestingly, various adipose-derived factors were reported to play a role in MSC-mediated pro-tumorigenic effects. For instance, adipokine chemerin is a major player in obesity-mediated support of cancer progression. This cell differentiation promoter and leukocyte chemoattractant factor was reported to promote the growth, proliferation migration, invasion, and metastasis of cancer cells *via* the recruitment of tumor-associated MSCs and the stimulation of angiogenesis pathways in endothelial cells through chemerin receptor 1 (CMKLR1), chemerin receptor 2 (GPR1), and CCLR2 signaling^103^.

### Reprogramming microenvironment cells for anti-cancer effects

Early studies addressing the immunological hallmarks of MSCs in the tumor microenvironment revealed various molecular mechanisms through which MSCs modulate the immune response in the cancer microenvironment and indicated that it may be possible to convert the microenvironment from immunosuppressive to immunostimulant^104,105^.

*In vitro* studies support the anti-tumor effects of MSCs, but these effects can be markedly reduced *in vivo* by the tumor-trophic properties of these cells and the direct cell-to-cell integration with tumor stromal elements. A score of recent reports suggests promising strategies for reprogramming microenvironmental cells to mediate only anti-cancer effects. For instance, unlike conditioned medium from human adipose MSCs, eicosapentanoic acid-treated adipose MSCs reduce mRNA levels of the tumor-associated genes *FASN*, *STAT3*, and *cIAP-2* in MDA-MB-231 and MCF-7 breast cancer cell lines, with marked decreases in their glycolysis, inflammation, and motility *in vivo*^106^.

Mandal and colleagues^107^ proposed the encapsulation of MSCs from the perinatal tissue with the sodium alginate biomaterial. The team isolated the 3D structure from the microenvironment and observed that the encapsulated MSCs displayed: (i) increased proliferation with expression enhancement of pluripotency genes, EMT, immune-modulation, and angiogenesis; (ii) increased secretion of VEGF, TGF-β, TNF-α, IFN-γ, IL-10 and IL-6, and IL-3β; (iii) and increased expression of the tumor invasion suppressor protein E-cadherin^107^. Furthermore, treatment of CSCs derived from MDA-MB-231 and MCF7 breast cancer cell lines with encapsulated MSCs lowered CSC viability and migration, with downregulation of markers related to angiogenesis, EMT and proliferation, and upregulation of Wnt antagonists secreted frizzled-related protein 4 (sFRP4) and DKK1^107^.

Prolonged culture of heterogeneous prostatic CAFs resulted in a marked decrease in the expression of proliferative endothelial cell surface marker endoglin (CD105), as compared to short-culture CAFs, and loss of their tumor expansion potential and heterogeneity in 3D cultures and patient-derived xenograft tissues^108^. Engineered human placenta-derived MSCs, armed with a double fusion gene containing the herpes simplex virus truncated thymidine kinase and firefly luciferase, inhibited the tumorigenesis mediated by the HT29 colon cancer cell line in nude mice^114^. Treatment with 5-azacytidine restored IL-6-increased production in MSCs from myelodysplastic patients^116^.

A study addressing the response of human MSCs to low-dose photodynamic therapy revealed that this treatment may increase MSC immunogenicity and promote angiogenic potential^117^. In this *in vitro* study, low-dose photodynamic therapy: (i) induced the reorganization of MSC cytoskeleton, with a decrease in cell motility; (ii) induced the inhibition of glycogen synthase kinase-3 (GSK-3) and the activation of extracellular signal-regulated protein kinases 1 and 2 (Erk1/2) signaling in MSCs; (iii) significantly upregulated the secretion of VEGF-A, IL-8, plasminogen activator inhibitor-1 (PAI-1), MMP-9, and other proangiogenic factors by MSCs; (iv) dramatically inhibited the secretion of pro-tumorigenic macrophage infiltration marker CCL2 (MCP-1) by MSCs and decreased MSC viability and immunogenicity when cocultured with lymphocytes. In another study, MSCs loaded with a photosensitizer successfully shipped these nanoparticles into lung cancer tumor sites, enhancing the effects of photodynamic therapy *in vivo*^110^. In addition, irradiated endothelial cells decreased the malignancy of liver cancer cells in a coculture system using medium conditioned with endothelial cells, thus suggesting that irradiated endothelial cells are key players in the therapeutic effects of radiotherapy^115^. In addition, various flavonoids and non-flavonoid polyphenolic compounds from medicinal plants alleviate multidrug resistance in breast, prostate, lung, and colorectal cancer with survival benefits in patients, through their antioxidant capacity, the modulation of inflammatory responses, and the inactivation of oncogenes with the inhibition of survival, angiogenesis, proliferation, and metastasis^118^ (**Table 1**).

**Table 1:** Methods proposed for reprogramming the tumor microenvironment

Cancer type/model	Methods	References
Breast cancer	Treatment of MSCs with eicosapentanoic acid	^106^
	Encapsulation of MSCs with sodium alginate	^107^
Prostate cancer	Extended passaging of CAFs	^108^
	Elimination of tumor immunosuppressive cells with chimeric protein IL2-R336A	^109^
Lung cancer	MSCs loading with the photosensitizer MnO2@Ce6	^110^
	MSCs loading with nanoparticles	^111^
Hepatocellular carcinoma	MSCs carrying an adenovirus	^112^
	Treatment of MSCs with melatonin	^113^
Colon cancer	MSCs arming with a double fusion gene containing the herpes simplex virus truncated thymidine kinase and firefly luciferase	^114^
Liver cancer	Irradiation of endothelial cells	^115^
Neuroblastoma	Autologous MSCs carrying an oncolytic adenovirus	^75^
Myelodysplastic syndrome	Treatment of MSCs with 5-azacytidine	^116^

## Some causes of controversies on the roles of stromal cells in cancer

### Cancer cell lines

**Figure 1:**
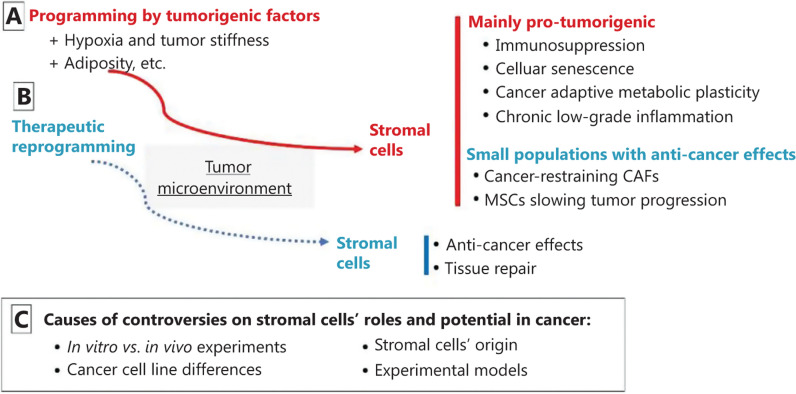
Summary of pro-tumorigenic (A) and therapeutic potential (B) of mesenchymal stromal cells (MSCs) and causes of controversies (C). CAFs, cancer-associated fibroblasts.

In numerous reports, it is not clear whether the pro-tumorigenic rather than anti-cancer role of MSCs is dictated by a cell line-specific event. In a coculture study with bladder cancer cells displaying stem cell-like properties (CD133+) and adipose-derived MSCs, the latter cells produced soluble mediators that: (i) increased the phosphorylation of molecules involved in cancer progression and drug resistance, such as p70 S6K, ERK1/2, and AKT1/2/3 in CD133+ cells from 5637 cell line; but also (ii) decreased the phosphorylation of those PI3K/Akt and MAPK signaling molecules in CD133+ cells from HB-CLS-1 cell line^119^. MSCs in fact induced pro-tumorigenic effects in the presence of 5637 bladder cancer cell line and anti-cancer effects in the presence of HB-CLS-1 bladder cancer cell line, and thus the effect of crosstalk between MSCs and bladder cancer cells remains unclear. Similarly, in a study assessing how breast cancer cells from different stages of the metastatic cascade convert MSCs into tumor-associated MSCs, only MDA-MB-231 breast cancer secretomes, but not MCF-7 cells and sublines isolated from bone, lung, and brain metastases, converted MSCs into tumor-associated MSCs in bioengineered 3D microenvironments^120^. Altogether, these findings suggest that MSCs from the tumor microenvironment are pre-conditioned to mediate pro-tumorigenic effects on cancer cells, and that impeding this pre-conditioning or re-conditioning MSCs may warrant anti-cancer effects in the tumor microenvironment (**Figure 1**).

### *In vitro vs. in vivo* and MSC origin

Experimental evidence supports the idea that stromal cell effects and origin may explain the discrepancies amongst data from *in vitro* and *in vivo* studies. Quach and colleagues^121^ reported that while the inhibition of the glypican-1 (GPC-1) prostate cancer biomarker decreases cell growth and migration *in vitro* of the aggressive prostate cancer cell line PC-3, quite surprisingly it increases the PC-3 tumor size in NCr nude mice xenografts. Also surprisingly, it increases cancer cell proliferation and migration in aggressive prostate cancer cell line DU-145 cells, suggesting that GPC-1 accounts for among the factors that drive a cell line-dependent response to stromal cells. In addition, in the same study, the decreased cell growth observed in GPC-1 knockdown PC-3 cells was rescued by coculturing the cells with MSCs and CAFs. Further, treatment of these stromal cells with tumor-conditioned media from PC-3 cells transfected with GPC-1 short hairpin RNA (shRNA) increased the expression of ECM components, endocrine and paracrine biomolecules, and migration markers^121^. Moreover, despite *in vivo* observations suggesting the ability of this signaling pathway to induce drug resistance and influence the ability to form metastasis *via* induction of EMT in pancreatic cancer, the activation of insulin-like growth factor (IGF)/IGF-I receptor (IGF-IR) signaling by stromal cells failed to induce EMT in cultures with MiaPaCa-2, AsPC-1, Capan-2, BxPC-3, and Panc1 pancreatic cancer cell lines^122^.

Considering that MSCs promoted anti-cancer effects in most reports, as discussed in the Cancer-restraining CAFs section, surprisingly, treatment of MDA-MB-231 and MCF-7 human breast cancer cells with medium containing extracellular vesicles promoted the *in vitro* proliferation and migration of cancer cells through ERK signaling^123^. We hypothesize that these effects may be due to differences in the origin of MSCs, as in this study human umbilical cord MSCs, and not BM or adipose-derived MSCs were used. As a further support of this hypothesis, a comparative study of subcutaneous and visceral adipose-derived MSCs revealed various functional similarities and differences, despite similar surface markers^124^. Notably, visceral MSCs secreted higher levels of inflammatory cytokines (IL-6, IL-8, and TNF-α) and had a more active sonic hedgehog pathway than subcutaneous MSCs. Fetal and adult lung MSCs possess lung-specific properties, unlike BM-MSCs^22^. However, a study profiling the transcriptomes of 361 single MSCs derived from two umbilical cords (UC-MSCs), harvested at different passages and stimulated with or without inflammatory cytokines, revealed that UC-MSCs are a well-organized population with limited heterogeneity, as compared to other MSC types^125^.

## Conclusions

The available data clearly support that stromal cells normally have anti-cancer effects, and that reprogramming by cancer cells in the tumor microenvironment induces their switch to pro-tumorigenic activities, thus suggesting that targeting the tumor microenvironment could be a promising therapeutic strategy in cancer. A growing number of reports suggest the possibility to reprogram stromal cells to maintain or revert back to anti-cancer effects. Interestingly, cancer-restraining stromal cells have been identified in the microenvironment, and a marker was reported. Future studies characterizing the origin of these cells may provide clues to how they can be exploited for anti-cancer therapy. The emerging data shed light on the origin of previous controversies on the roles of stromal cells in the tumor microenvironment. Notably, MSCs have varying effects on cancer cell lines of different origins, and MSCs from different origins have different effects on cancer cell cocultures. On the other hand, the tumor microenvironment induces complex signals that affect how stromal and cancer cells respond to soluble factors *in vitro* and *in vivo*. These discrepancies should be taken into account in the design of future studies and interpretation of results.
